# Pulmonary hypertension: role of septomarginal trabeculation and moderator band complex assessed by cardiac magnetic resonance imaging

**DOI:** 10.1186/1532-429X-11-S1-P91

**Published:** 2009-01-28

**Authors:** Monda L Shehata, Jan Skrok, Dirk Lossnitzer, Danielle Boyce, Joao AC Lima, Paul Hassoun, David A Bluemke, Jens Vogel-Claussen

**Affiliations:** 1 Johns Hopkins University, Baltimore, MD USA; 2 University of Heidelberg, Heidelberg, Germany; 3 National Institute of Health (NIH), Bethesda, MD USA

**Keywords:** Pulmonary Arterial Hypertension, Right Ventricle, Idiopathic Pulmonary Arterial Hypertension, Pulmonary Arterial Hypertension Patient, Pulmonary Hemodynamic

## Introduction

In the right ventricle (RV), the septomarginal trabecula (SMT) arises as a muscular band originating from the interventricular septum (IVS) at the lower segment of the crista supraventricularis. It forms a functional unit with the moderator band, which attaches to the lateral free wall of the RV [1, 2]. Strategically situated between the RV inflow and outflow tracts, the whole unit serves to help emptying blood into the pulmonary trunk during systole. Thus, it should be anticipated that the SMT may undergo changes in RV hypertrophy secondary to chronic pulmonary hypertension.

## Purpose

To assess SMT mass in pulmonary arterial hypertension (PAH) and its relationship with RV function and pulmonary hemodynamics using cardiac MRI.

## Methods

Imaging was performed in two centers; one using 3 T and the other using 1.5 T MR systems. 33 catheter proven PAH patients (mean age = 61.4 ± 12.1 years and mPAP = 45.9 ± 12.4 mmHg) were enrolled in the study [Idiopathic pulmonary arterial hypertension (IPAH) = 21 and Scleroderma (SSc) = 12 patients]. Similarly, 9 healthy volunteers (mean age = 45.56 ± 8.6 years) were included in the study for comparison. Short axis cine images were acquired using fast gradient echo technique. End diastolic frames were analyzed using MASS 6.2.1 software (Medis, the Netherlands). Starting from the basal slices, the SMT was identified in patients and controls as the most anterior trabeculation arising from the IVS below the outflow tract level. Two independent observers manually contoured and traced the SMT from its origin towards the apex where the moderator band and secondary trabeculation arise. SMT mass in grams was derived from the volume based on a myocardial density of 1.05 g/cm^3^. Epicardial and endocardial ventricular borders were semi-automatically contoured for quantification of ventricular mass and functional indices. Ventricular mass index [ratio of RV over left ventricular (LV) mass] was derived for all groups.*The Mann-Whitney* test was used for direct comparisons. Correlation between SMT mass and MR derived cardiac functional indices as well as catheter derived hemodynamic parameters was tested using *Spearman's rho* correlation.

## Results

**Figure 1:**
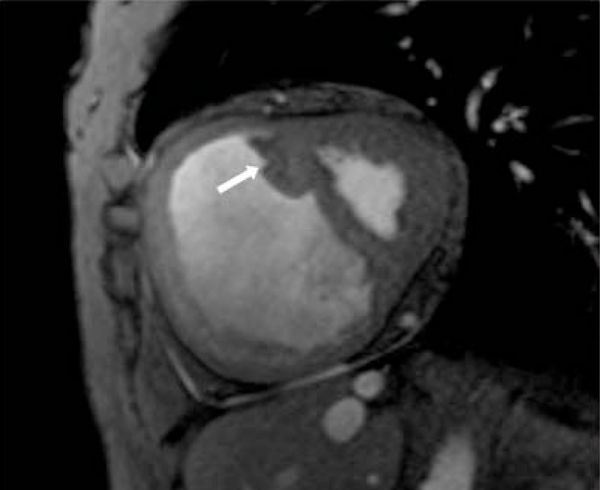
**64-year-old female with mean PAP = 51 mmHg demonstrates hypertrophy of SMT (septomarginal trabecula), white arrow, in a dilated right ventricle**.

In all subjects, the SMT was observed in the RV; however, the morphology was highly variable. The SMT mass was greater in PAH patients compared to controls, 5.0 ± 3.9 g and 1.2 ± 0.7 g respectively (P = 0.0001). Similarly, significant differences in SMT mass were also noted between controls and each subgroup: IPAH (p < 0.001) and SSc (p < 0.01). In PAH patients, SMT mass showed significant correlations with catheter derived pulmonary hemodynamics mPAP (r = 0.4, p = 0.032) and PA systolic pressure (r = 0.4, p = 0.01). It also demonstrated good positive correlations with RV end diastolic mass indexed to body surface area (RVED mass/BSA r = 0.5, p = 0.006), VMI (r = 0.56, p = 0.001), RV end systolic volume/BSA (r = 0.4, p = 0.01) whereas, it correlated inversely with RV stroke volume (r = -0.5, p = 0.003) and RV ejection fraction (r = -0.6, p = 0.0001). SMT mass displayed stronger correlations with IPAH RV structural and functional indices compared to patients with Scleroderma. Interobserver concordance for SMT mass quantification was r = 0.95, (p = 0.0001). Figures 1, 2 and 3

**Figure:**
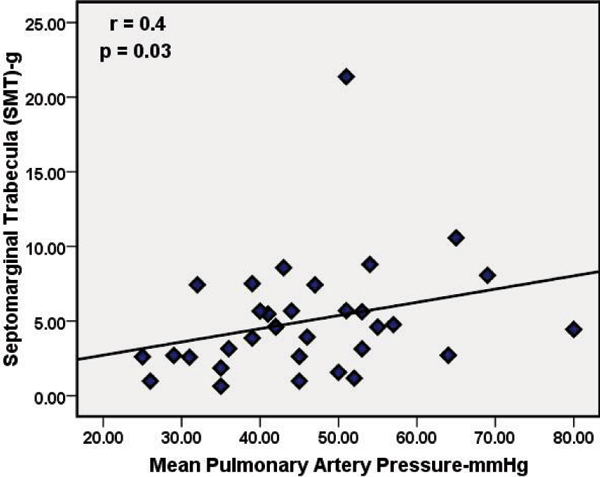
Figure 2

**Figure:**
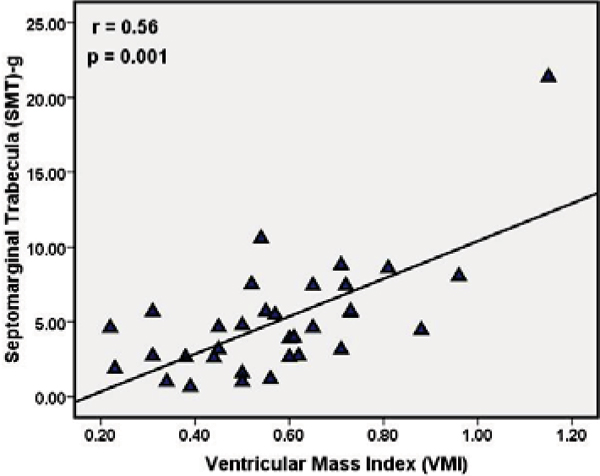
Figure 3

## Conclusion

SMT/moderator band complex hypertrophy was seen in all PAH patients studied. Degree of SMT hypertrophy correlated with RV systolic function and pulmonary hemodynamics. The SMT is a unique RV anatomical structure which contributes to RV adaptation mechanisms in chronic PAH. It is readily assessed with cardiac MRI and aides in the diagnosis of PAH.
